# A Comprehensive Review on Seaweed Preservation Techniques: Impacts on Nutritional Quality, Safety and Shelf Life

**DOI:** 10.1002/fsn3.72172

**Published:** 2026-09-07

**Authors:** Shanjida Islam Shikha, Hrishika Barua, Md. Faisal

**Affiliations:** ^1^ Department of Fishing and Post‐Harvest Technology Chattogram Veterinary and Animal Sciences University Chattogram Bangladesh

**Keywords:** drying of seaweed, seaweed, seaweed acceptance, seaweed preservation, seaweed shelf life

## Abstract

Seaweeds are important marine resources that contain a great variety of beneficial compounds, including protein, essential fatty acids, vitamins, minerals, fibers, and bioactive compounds such as polyphenols and carotenoids. Nutritional, functional, and industrial properties of these marine algae make them highly applicable to culinary, nutraceutical, pharmaceutical, agricultural, and cosmetic sectors. Seaweeds are perishable as their quality gets highly influenced under certain conditions, such as high moisture content, enzymatic activity, and microbial presence. Parameters such as water activity, lipid indices, total volatile base nitrogen, microbial load, pathogen detection, pH, and sensory properties are used to determine the loss of quality and product stability over time, helping in the determination of the shelf life of seaweed during the post‐harvest storage period. To extend the shelf life, several preservation methods have been explored. Traditional methods include sun drying, oven drying, salting, and fermentation, which provide easier and lower‐cost conditions, but sometimes cause a compromise in quality. Modern techniques like high‐pressure processing, pulsed electric fields, modified atmosphere packaging, and irradiation appear as solutions with lower loss of quality, higher nutrient retention, microbial safety, and sensory attributes. This review critically evaluates post‐harvest deterioration mechanisms, quality assessment parameters, traditional and advanced preservation techniques, their impact on shelf life and product quality, and current consumer perceptions to promote sustainable utilization of seaweeds in response to rising global demand.

## Graphical abstract

This review demonstrates essential quality assessment metrics for monitoring product stability and identifying the major causes of post‐harvest deterioration of seaweed. It evaluates common and advanced preservation techniques for their ability to extend shelf life while maintaining nutritional value, microbiological safety, and sensory quality. This can promote sustainable use of seaweeds, ensure consumer acceptance, and support a growing market trend.

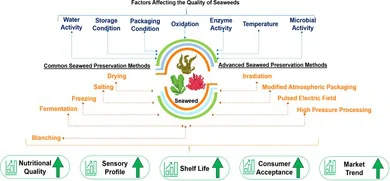

## 1 Introduction

Seaweeds are recognized as one of the most promising resources of the 21st century, offering substantial environmental, social, and economic benefits. 97% of the world's seaweed production, or 35.8 million tons, is currently produced through aquaculture, and the global market is worth $11.8 billion (Sultana et al. 2023). By 2027, it is expected that the seaweed market will be worth $12.85 billion worldwide (Ismail and Zokm 2025). In addition to being a source of nutrient‐dense human food and byproducts, seaweed farms also help to mitigate climate change by absorbing carbon, acting as a sink for CO_2_ (Krause‐Jensen and Duarte 2016), and lowering agricultural emissions by supplying raw materials for the production of biofuel and animal feed (Duarte et al. 2017). Generally, seaweeds have the capacity to absorb enough carbon to elevate water pH (Campbell et al. 2019), lessen the consequences of ocean acidification and hypoxia (Duarte et al. 2017). Additionally, seaweed farming has been seen as a strategy to empower coastal women in the majority of low‐income and developing nations (Sultana et al. 2023). Also, it contributes to the blue economy and is thought to have less of an environmental impact than some other aquaculture techniques, like shrimp and finfish culture (Eggertsen and Halling 2021).

Few industries on the East Coast of the United States of America (USA) and Canada have started cultivating seaweed, especially for human consumption. Food products and cookbooks with recipes utilizing “sea vegetables” are also sold in many countries across the world (Mouritsen et al. 2019). The need for new meals that satisfy the demands of an expanding population and also offer some health benefits has been spurred in recent decades (Granato et al. 2020). Approximately 66% of algae species have long been utilized as a daily ingredient in food in nations like China, Korea, and Japan (Rajapakse and Kim 2011). Apart from these countries, seaweeds are vastly utilized in other American nations, such as Mexico, which has continued to incorporate algae into its cuisine. On the other hand, the use of seaweed in the daily diet is less common in nations like Spain and Portugal, even after having long coastlines and a large variety of species. However, in recent years, seaweed has been recognized as an important nutrient source that can be added to the diet, particularly for people who are vegetarians or vegans (Peñalver et al. 2020).

Seaweeds are gaining commercial importance due to various bioactive compounds and secondary metabolites that are present in seaweeds, which can be further used in the food, pharmaceutical, nutraceutical, fertilizer, and cosmetics industries (Pradhan et al. 2022). Additionally, seaweeds are sources of commercially valuable hydrocolloids such as agar, agarose, carrageenan, and alginate, thus increasing their economic value (Jeeva et al. 2012). Because of the potential utilization of these substances, seaweed quality must be preserved after harvest to ensure its suitability for further industrial and food applications. Traditionally, the quality of seaweed has been evaluated by people using sensory analysis (appearance, color, odor, texture, etc.) (Sánchez‐García et al. 2021). However, with technological advancements, physicochemical (protein, lipid, moisture, ash, fiber, and pH) and microbiological analyses are also considered quality parameters for seaweeds exposed to different heat treatments and storage conditions (Blikra et al. 2019). Microbiological assessment typically includes total viable counts (TVC), while advanced techniques such as multispectral imaging (MSI), FT‐IR spectroscopy, and electronic nose (e‐nose) systems are increasingly applied to improve accuracy and efficiency (Lytou et al. 2022).

Appropriate preservation methods are crucial to maintain seaweed quality from harvest to final processing. Among traditional preservation techniques, sun‐drying is commonly used because it is inexpensive and simple (Robic et al. 2008). Although drying offers many benefits, studies have reported that drying technologies can reduce bioactive compounds, including total phenols, flavonoids, and vitamin C, even at low drying temperatures (30°C, 25% humidity, 3.5 h) (Sappati et al. 2019). To avoid this, low‐temperature preservation methods, such as freeze‐drying and freezing, are more effective at preserving functional quality than other methods. Furthermore, compared with hot‐air drying, brining, and dry‐salting stabilization, freeze‐drying *Ulva lactuca* (formerly *Ulva rotundata*) (Chlorophyta) and storing the extracted ulvan polysaccharide at −30°C and −80°C resulted in ulvans with larger molecular weights (Robic et al. 2008). These high‐molecular‐weight ulvans exhibit better water retention and texture, along with reduced degradation (Hussein et al. 2015; Kansandee et al. 2024), which can be further utilized for stable gel processing (Souza et al. 2023).

**FIGURE 1:**
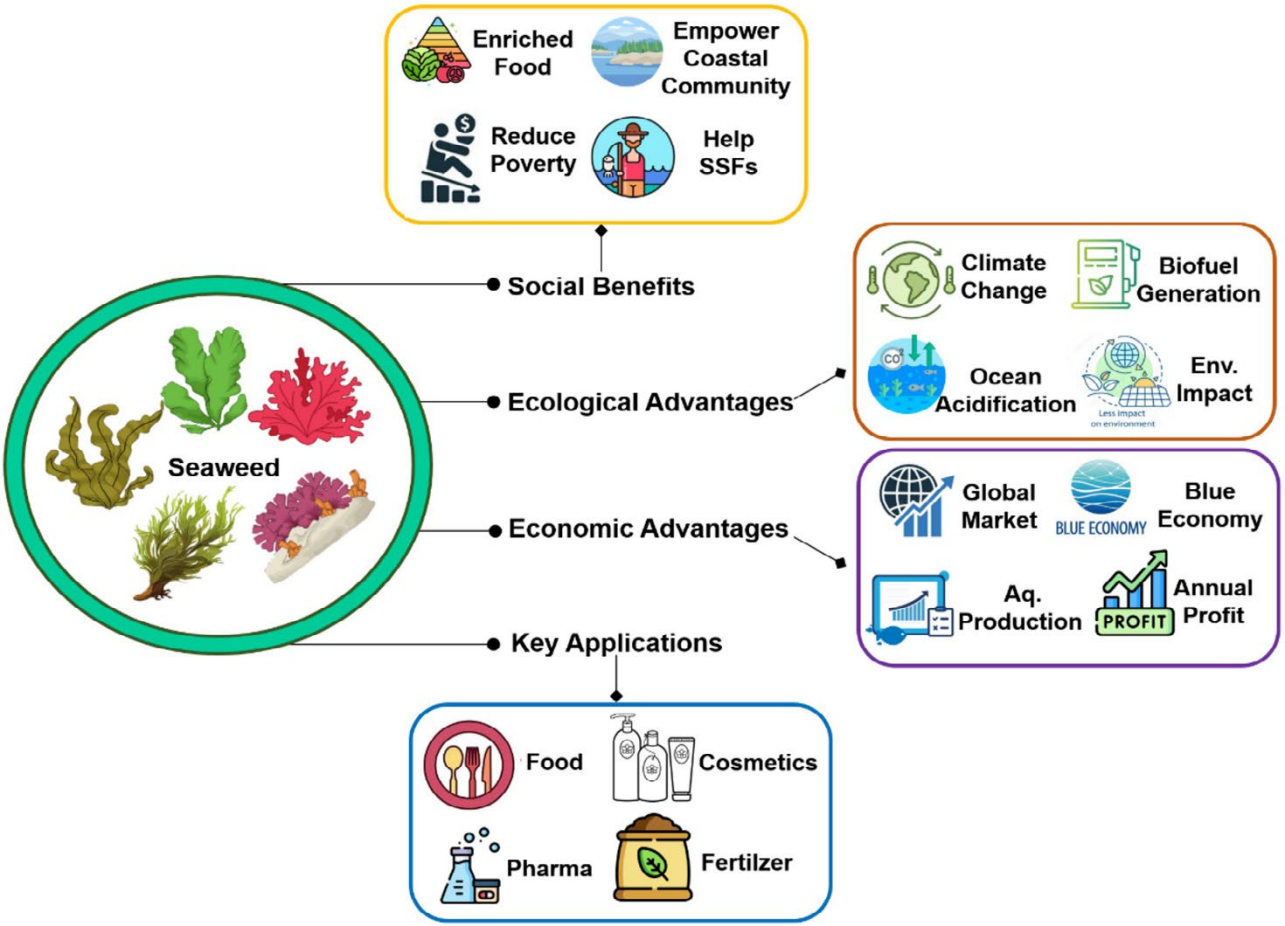
Summary of advantages and uses of seaweed for quality improvement and preservation.

Though seaweeds have multifaceted benefits, FAO reported some of the risks linked with seaweed consumption, which can lead to death in some rare cases. These risks include arsenic (As), cadmium (Cd), dioxins, radionuclides, pesticides, virus (norovirus), *Salmonella* spp., *Shigella* spp., metal, glass, microplastics, and allergies (Cherry et al. 2019). These risks can be minimized by appropriate processing and preservation methods. Understanding how the seaweed contents change in response to various preservation strategies is essential for incorporating seaweeds into a wide range of products. Consumer acceptability and sustainability goals have raised the pressure. To address these issues, this review looks at the importance of seaweed in relation to quality standards, preservation methods, and consumer demand. By doing so, it will be easier to suggest future research directions and commercial applications for the exploitation and preservation of seaweed (Figure 1).

## 2 Methodology

The comprehensive review was done through a broadened narrative method that aimed at encompassing the scope and profundity of the current research surrounding different seaweed preservation techniques and their impacts on nutritional quality, safety, and shelf life. To conduct the review, articles, both research and review, published between November 2008 and June 2025 were studied. Electronic databases, such as Google Scholar, were used to search using an integration of several keywords (Table 1). Potentially eligible studies were filtered based on pre‐defined inclusion criteria, which focused on peer‐reviewed articles, conceptual relevance, methodological clarity, and full‐text availability, whereas non‐scholarly articles, incomplete reports, and those that were not directly relevant were discarded. The information about each of the included studies was copied onto a structured template, including authorship, year of publication, study design, sample, analysis methods, and main findings. The quality of the study was evaluated in terms of study design, methods of analysis, and reliability of data. The initial screening process included the title and abstract review, which was followed by examination of full‐text articles. Key thematic areas were carefully reviewed to determine the actual situation. The review was done by reviewing each article in terms of objectives attained, the validity of the methods used, the correctness of data analysis, and its applicability to seaweed consumption. The articles which lacked methodological soundness (such as those that had a small sample size, non‐reproducible methodology, or ambiguous results) were filtered out. To achieve consistency and minimize redundancy of the collected data, cross‐referencing was used.

**TABLE 1:** Keywords used to conduct this review article.

Section	Keywords
Introduction	Seaweed and the global economy, seaweeds in human consumption, benefits of seaweed, post‐harvest treatment of seaweeds, preservation strategies for seaweed, seaweed in relation to quality standards, preservation methods, and consumer demand, etc.
Nutritional importance of seaweed	Seaweeds as a natural source, nutritional profile of seaweeds, health benefits of seaweeds, industrial applicability of seaweeds, bioactive compounds in seaweeds, seaweeds consumption level, etc.
Quality evaluation parameters of seaweed	Microbial quality in seaweed, methods of evaluation of shelf life, alterations in the quality of postharvest seaweeds, effects of seaweed storage temperatures, quality indicators on a color basis, physicochemical parameters of seaweed, conservation and stability of seaweeds, technologies of quality monitoring, etc.
Factors affecting the quality and shelf life of seaweed	Quality of post‐harvest seaweed, stability of shelf‐life of seaweed, storage temperature impact on seaweed, moisture content impact on seaweed, bacterial degradation of seaweed, factors affecting seaweed longevity, seaweed degradation through oxidation, packaging effect on shelf‐life, seaweed sensory properties degradation, qualitative environmental factors, etc.
Shelf life of seaweed	Storage conditions and shelf‐life, preservation impact on seaweed, quality retention in seaweed, etc.
Common preservation techniques of seaweed	Common methods of preserving seaweeds, seaweed drying methods, salting of seaweed products, preservation of seaweed through fermentation, sun‐drying of marine algae, seaweed storage practices, natural conservation of algae, etc.
Advanced preservation techniques of seaweed	Advanced methods of seaweed preservation, applications in high‐pressure processing, pulsed electric field treatment, and seaweed modified atmosphere packaging. New food preservation methods, advanced storage systems for seaweed, preservation in thermal and non‐thermal conditions, etc.
Consumer acceptance & market trends of seaweed	Consumer preferences of seaweeds, market trends in seaweed, seaweed product acceptance, seaweed demand and supply, seaweed products popularity, trends in seaweed in the food industry, factors in the growth of the seaweed market, etc.

## 3 Nutritional Importance of Seaweed

Seaweeds are recognized as a natural source of various bioactive compounds, vitamins, minerals, proteins, lipids, carbohydrates, carotenoids, polyphenols, and enzymes (Matos et al. 2024). Seaweed proteins are considered important because, depending on species and environmental conditions, they contain a relatively wide range of protein (5% to 47%) of dry weight (DW). These proteins are enriched with both essential (especially lysine, leucine, isoleucine, phenylalanine, valine, etc.) and non‐essential amino acids (glycine, alanine, proline, glutamic, and aspartic acids, etc.), which are necessary in the human diet. In seaweed, essential amino acids make up over half of all amino acids, and the protein composition is similar to that of egg protein (Černá 2011). Therefore, seaweed is regarded as a valuable plant‐based protein source, particularly for those on vegetarian or vegan diets. By adding seaweed to the diet, one can help to fulfill daily protein needs and give additional health benefits related to its nutrient content (Raja et al. 2022). Among three different classes of seaweeds, green and red seaweeds generally have higher protein contents (10%–47% DW) than brown seaweeds (5%–24% DW). Although the range of lipid content is low (0.5%–4.5% DW) in seaweed (Schmid et al. 2018), they are essential to nutrition because the availability of *ω*‐3 and *ω*‐6 polyunsaturated fatty acids (PUFAs) makes up a substantial portion of the seaweeds' lipid profile (from 0.79% to 7.87% dry matter) (Pereira 2018). Green and red seaweeds exhibit very low lipid concentrations, ranging from 0.3% to 2.1% DW, whereas brown macroalgae have slightly higher values, ranging from 1.8% to 4.8% DW. Despite intergroup differences, several macroalgal species have been shown to have comparable lipid ranges (García‐Poza et al. 2020). The fatty acid composition also differs among algal groups, such as EPA, palmitic acid, oleic acid, and arachidonic acid, which are generally higher in red seaweeds than in brown seaweeds. Brown seaweeds have low EPA but high levels of oleic acid, linoleic acid, and α‐linolenic acid. DHA, α‐linolenic, palmitic, and oleic acids are all more abundant in green seaweeds (Kumari et al. 2010). Among the species studied by García‐Poza et al. (2020), *Chondrus crispus* (Rhodophyta) has the highest levels of carbohydrate content, accounting for about 70% of its biochemical components. Again, seaweeds contain both water‐soluble and fat‐soluble vitamins that contribute to their nutritional value (Finglas et al. 2015). Water‐soluble vitamins of seaweed possess especially B1 (thiamine) and B12 (cyanocobalamin), while fat‐soluble vitamins possess vitamin A and vitamin E (from tocopherols) (Škrovánková 2011). Seaweeds are also known for their high mineral content (8%–40% DW) due to their ability to directly absorb dissolved elements from seawater across the thallus, resulting in higher mineral accumulation than in many land‐based plants, although levels vary with species, season, and environmental conditions (Peñalver et al. 2020). As a result, seaweeds are a good source of macro (Na, K, Ca, Mg) and micro (Fe, Zn, Mn, Cu, I) minerals (Lozano Muñoz and Díaz 2020). However, consumers should be careful while eating seaweeds as certain seaweeds contain higher amounts of iodine (García‐Vaquero and Hayes 2016). For all species, a maximum of 2000 mg kg^−1^ DW of iodine is advised in France. However, pregnant women, those with heart or kidney disorders, and those with thyroid disorders (who also take iodine‐related medications) should avoid consuming iodine or its derivatives (Salido et al. 2024). In addition to the nutritional value, there have been reports of bioactive compounds in macroalgae with significant industrial applicability, and numerous uses are now under development. The pharmaceutical industry has utilized them for their anti‐inflammatory, antimutagenic, anticancer, antidiabetic, and antihypertensive properties, as well as in the food, fuel, polymers, and cosmetics sectors (Alisha and Haider 2019; Mohammed et al. 2021). The variability in nutrient profiles among seaweed species supports the need to select preservation methods according to their major biochemical components, since such differences can influence seaweed quality during storage.

## 4 Quality Evaluation Parameters of Seaweed

Techniques commonly used in the quality evaluation of sea vegetables and other marine products included microbiological counting, sensory assessment, chemical parameters (total volatile base nitrogen (TVB‐N) and trimethylamine (TMA‐N)), and physical measures (*a*_*w*_, pH). In freshly stored seaweed, microbial load, volatile bases, pH, and sensory parameters are found to be more reliable quality parameters. For example, TVB‐N and TMA‐N of *Ulva rigida* (Chlorophyta) did not grow until 10 and 6 days (shelf life), at 4°C and 16°C, respectively. This is consistent with the pH rise caused by the synthesis of basic chemicals. Similarly, microbial deterioration and the production of amines and volatile bases also started when the population reached 4.0 × 10^8^ mL^−1^ and 2.5 × 10^8^ mL^−1^ microbial cells at 4°C and 16°C, respectively, influencing the quality (Sánchez‐García et al. 2021). While manufacturing a final product for consumers, food manufacturers must take into account different visual characteristics like color, odor, etc. For instance, following 3 days of storage at 4°C, the color properties of *Ascophyllum nodosum* (Phaeophyceae) were L* (28.58 ± 1.41), a* (0.51 ± 0.19), and b* (3.08 ± 0.92). These were evaluated as a*, b*, and L* values, used as indicators, and fluctuated somewhat after storage, indicating quality alterations (Zhu, Patange, et al. 2022). These findings suggest that TVB‐N, TMA‐N, microbial load, and color changes should be prioritized when evaluating the quality of refrigerated seaweed.

For dried seaweed products, moisture control is crucial as commercial dried seaweed is usually kept at a relative humidity (RH) of 90% and 25°C for 15 days had mold/yeast populations of log 6.42 CFU/g; however, these populations were much lower when kept at RH 70% (log 2.12 CFU/g) and 50% (log 1.35 CFU/g) (Hyun et al. 2018). To guarantee food safety, the general administration of quality supervision, inspection, and quarantine in China (AQSIQ) declared that mold levels in dried laver products must be < 300 CFU/g (Choi et al. 2014). The relationship between moisture levels and microbial load emphasizes the importance of controlled storage conditions in preventing quality deterioration of dried seaweed products.

In processed seaweed, physical and sensory parameters change during storage and act as determinants of quality, like every group of kelp gel granule had an initial pH of about 9.35. Over the course of the storage period, there was little variation in the pH value of the items kept at 4°C. On the 20th day, however, the pH of the granules kept at 25°C was 6.96. Additionally, the items that were kept at 4°C lost some of their brightness and turned dark green instead of their original green tint. The products spoiled between 12 and 20 days, which led to a sharp decline in chewiness and hardness and an increase in cohesion and elasticity (Chen et al. 2024).

Seaweeds include distinct sensory qualities, including tastes of umami and salt, as well as flavors and odors that can be classified as marine, crustacean, or green, which can be used in food products with high potential (Jönsson et al. 2023). Fresh seaweed typically has a shelf life of 3 to 14 days (Lytou et al. 2021), depending on a number of factors like early or late harvest, pre‐ and post‐harvest treatments, and environmental conditions during the time leading up to harvest. Therefore, in addition to stabilizing the product to prevent food loss or waste, that is, spoiling, quick analytical methods are also required to estimate the microbiological quality and freshness. The profitability of the seaweed culture industry has increased, as well as sustainability, and higher‐quality products are marketed through these strategies. Consequently, stakeholders have worked very hard over the past few decades to find quick techniques that work for online, real‐time food safety and quality evaluations (Tsakanikas et al. 2020). Recently, new techniques, such as the use of e‐nose and e‐tongue systems in food quality evaluation, have been extensively studied, with a focus on widely used pattern recognition algorithms (Tan and Xu 2020). Multispectral imaging and Fourier Transform Infrared (FT‐IR) spectroscopy have also been studied for the assessment of various products of plant and animal origin (Karimi et al. 2016). According to Sánchez‐García et al. (2021), depending on the type of seaweed, some metrics did not help to identify changes in quality and required modifications.

Based on the reviewed studies, no single parameter is sufficient for assessing seaweed quality under all conditions. Considering variations in seaweed species and the postharvest storage conditions, more suitable techniques for determining seaweed quality and shelf life must be established.

## 5 Factors Affecting Quality and Shelf Life of Seaweed

**FIGURE 2:**
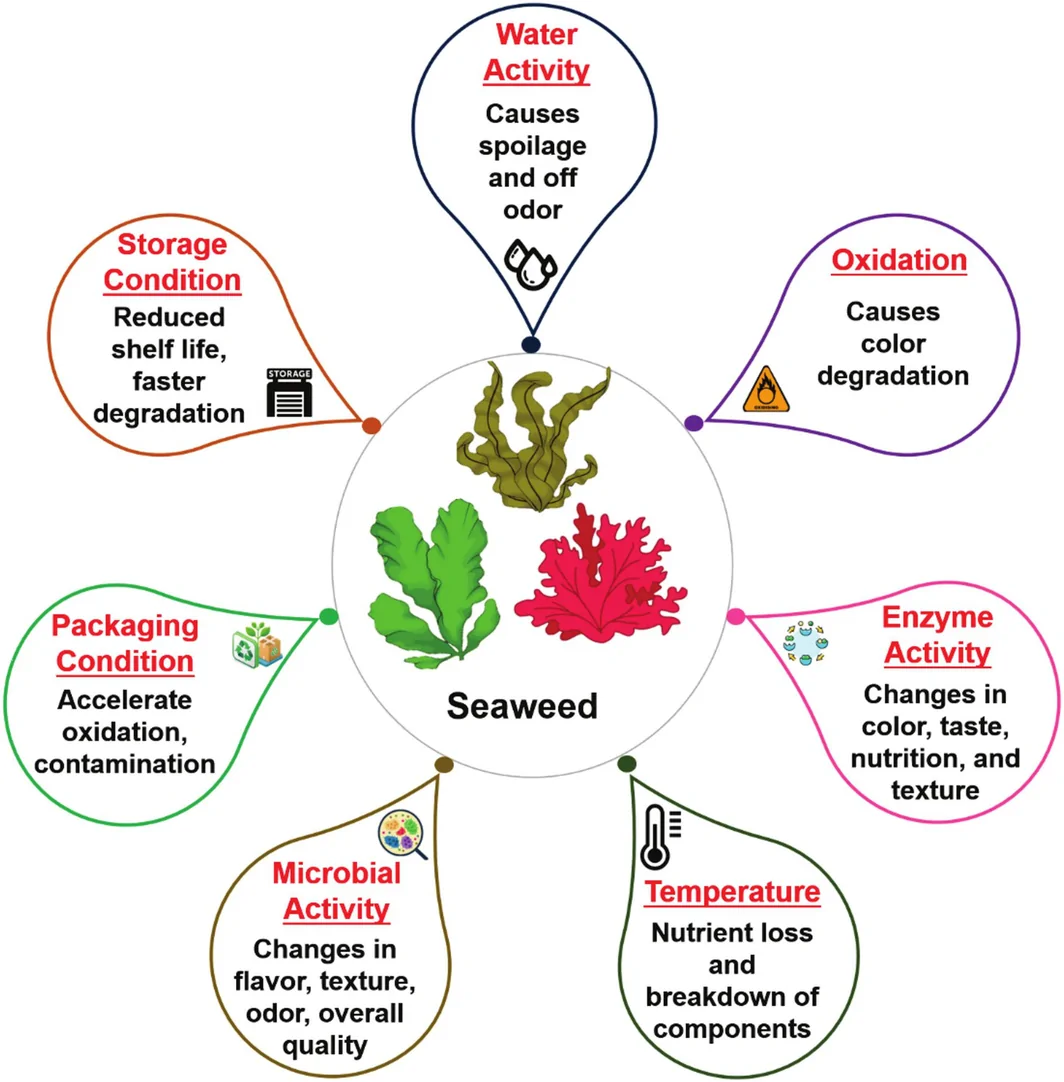
Principal factors influencing seaweed longevity.

Handling and processing procedures affect the quality of seaweed (Poeloengasih et al. 2019), as higher moisture content (Rasyid 2017) can affect shelf life and accelerate microbial development (Isharyadi et al. 2023). Mishandling and improper methods are typically the source of high moisture content in seaweed products (Santhoshkumar et al. 2023). Due to the high‐water (70%–90%) content, the quality of seaweeds deteriorates (Nayyar and Skonberg 2019), as a rich unsaturated fatty acid composition, oxidation sensitivity producing 4‐Hydroxy‐2‐hexenal/4‐Hydroxy‐2‐nonenal, near‐neutral pH, presence of non‐protein nitrogenous substances, and microbial activity are prone to significant quality reduction. Proper storage techniques are necessary to avoid spoilage (Rabiepour et al. 2024) (Figure 2).

### 5.1 Water Activity

The high moisture content and nutrient richness of seaweeds encourage microbial growth, making them perishable foods. Drying and salting are two traditional techniques for preserving seaweed that are based on lowering water activity (*a*_*w*_). Water activity (*a*_*w*_) in the environment is a typical way of characterizing the water requirements of microorganisms (Del Olmo et al. 2018). The ratio of the water vapor pressure of a food substrate to that of pure water at the same temperature is known as water activity. Water can dilute substrates, thus lowering reaction rates by acting as a solvent or reactant. It can also form complexes or hydrogen bonds with reacting species and control the mobility of reactants by altering the viscosity of food systems. Therefore, regulating water activity is an essential practical component for preventing unwanted enzymatic and chemical processes that might shorten food shelf life (Awulachew 2021).

In studies, *a*_*w*_ showed a major contribution in lipid oxidation and antioxidant degradation, where the oxidation and degradation increased significantly during storage at 40°C for 15 days at an *a*_*w*_ of 0.51 in dried laver (*Porphyra*) (Rhodophyta). The generation rates of hydroperoxides and CDA (conjugated dienoic acid) in dried laver lipids increased to 0.055 and 0.035, 0.128 and 0.083, 0.265 and 0.089, and 0.397 mmol/kg/day and 0.135%/day, respectively, when *a*_*w*_ climbed to 0.30, 0.51, 0.75, and 0.89. Lipid oxidation in dried laver was reduced by porphyran, α‐tocopherol, and polyphenols. After 15 days of storage at *a*_*w*_ of 0.11 and 0.30, respectively, the retention level of polyphenol in dried laver was decreased by 90.5% (7768.0 mg/kg) and 87.3% (7493.2 mg/kg). However, polyphenol degradation increased when dried laver was stored at *a*_*w*_ of 0.51, 0.75, and 0.89 for 15 days, leading to retention levels of 78.3% (6714.6 mg/kg), 70.0% (6009.6 mg/kg), and 67.0% (5749.4 mg/kg), respectively, influenced more by α‐tocopherol content than by polyphenols or porphyrin (Choe and Oh 2013). The changes suggest that the quality of dried seaweed products, such as *Porphyra*, can be maintained by minimizing *a*_*w*_ during storage while preserving tocopherol‐like antioxidant compounds.

### 5.2 Enzymatic Activity

A loss of texture at the sensory level results in higher softness, and a rise in stickiness reflects ongoing enzymatic and microbiological activity that breaks down the cell walls of the various seaweed components and the release of water during deterioration. The breakdown and decreased cell adhesion of polysaccharides in the cell wall are caused by enzymatic activities and water loss. This affects the structural elements that contribute to texture, resulting in a loss of crispness, hardness, and stiffness. In the initial days of storage, the seaweed softens and takes on a withered appearance. Textural profile study reveals lower consistency and resistance to compression (Sánchez‐García et al. 2021). According to Blikra et al. (2019), a change in seaweed color from fresh green to a duller green, and finally to yellowish‐brown, is a result of the molecules that break down chlorophyll and even the deterioration of the tissue itself, which is altered by enzymatic reactions and probably microbial activity.

Similarly, microbially derived enzymes can also accelerate some degradation processes. For example, recombinant *E. coli* that secretes the multifunctional amylase Amy19 demonstrated the capacity to immediately break down seaweed (*Gracilariopsis longissimi*—formerly *Gracilaria verrucosa* ) (Rhodophyta) trash. The concentration of reducing sugar rose steadily during the course of the degradation process, peaking at 60 h at 1.047 ± 0.0058 mg mL^−1^ (Zhao et al. 2023). This supports that alterations resulting in degradation, loss of texture, or appearance of seaweed can be caused by either an intrinsic or a microbially supplied enzyme.

### 5.3 Lipid Oxidation

Another important element affecting the stability of shelf life in seaweed products is lipid oxidation. Continuous lipid oxidation and the possible onset of rancidity were indicated by the TBA (thiobarbituric acid) value of a study, which rose dramatically from 0.02 to 0.19 malonaldehyde/kg sample. Apart from lipid degradation, fatty acids, ascorbic acid, and color changes were also noted in *Porphyra* and *Ulva* following oven‐drying at 40°C and during the 370‐day storage period in light, semi‐light, and dark settings (Salsabila et al. 2025).

Variation in drying and storage conditions also affects lipid oxidation. In an experiment, higher levels of lipid oxidation product in freeze‐dried (24 h) samples of *Porphyra* (*a*_*w*_ = 0.15/0.16) were noticed during storage of 520 days, compared to oven‐dried (40°C, 7 h and 40 min) samples (*a*_*w*_ = 0.20–0.29), which may be explained by the fact that lipid oxidation has a minimum rate at *a*_*w*_ ~ 0.3, below which the rate increases due to more efficient metal‐catalysis and hydroperoxide breakdown. Malondialdehyde (MDA), 4‐hydroxy‐2‐hexenal (HHE), and 4‐hydroxy‐2‐nonenal (HNE) were found to increase during storage, with 89%–96% PUFA loss under light settings. *Porphyra* had the highest HHE level of 2.4 μg/g at day 370, which contributed to color loss and quality deterioration (Harrysson et al. 2021), revealing the enhanced susceptibility of PUFA‐rich seaweeds to oxidative deterioration during storage.

### 5.4 Microbial Activity

High initial bacterial loads typically have a detrimental impact on the sensory quality and shelf life of products, but they may not always indicate that the food is hazardous to eat. Spoilage bacteria deteriorate the product but are not always dangerous to the customer. Bacterial growth conditions (Table 2) and environmental and processing contamination of raw materials are the primary causes of bacterial contamination. By lowering the water activity (*a*_*w*_) to 0.6 or less, drying can stop the growth of all microorganisms, including mold and yeast. Techniques for drying at high temperatures can be developed to render bacteria and their spores inactive. When dried seaweeds are used as ingredients in moist foods that will have a shelf life after the seaweeds are added, this could be interesting (Løvdal et al. 2021). A comparatively small number of pathogens, particularly the bacteria that produce toxins, can cause serious health issues or even death in humans. But during handling and processing, seaweed food items can potentially become polluted or re‐infected (Banach et al. 2020; Sakon et al. 2018).

**TABLE 2:** Factors influencing the quality of seaweed.

Factors	Deterioration process	Effects on quality	References
Water activity	Impact on microbial growth	Spoilage and off odor	Chitrakar et al. (2019)
Oxidation	Lipid oxidation causes deterioration	Changes in color	Salsabila et al. (2025)
Enzymatic activity	Protease, lipase, and oxidase reactions	Changes in color, taste, nutritional content, and texture	Blikra et al. (2019)
Microbial activity	Activity of bacteria, yeasts, and molds	Changes in flavor, texture, odor, and overall quality	Banach et al. (2020)
Packaging condition	Influence of oxygen and moisture permeability	Accelerated oxidation, contamination	Latief et al. (2019)
Storage condition	Temperature, humidity, and exposure to light	Reduced shelf life, faster degradation	Oh et al. (2014)
Temperature	Higher temperature leads to microbial growth and faster reactions	Higher nutrient loss	Nayyar and Skonberg (2019)

In a study, based on sensory analysis, the refrigerated (+2.8°C) shelf‐lives for sugar kelp from all treatments were determined to be 7 to 9 days. The end of the sensory shelf‐life correlated with the development of > 7 log (CFU/g) aerobic viable counts, suggesting this attribute can be used as a way to evaluate the shelf‐life of sugar kelp. After seven days, microbial communities in all samples increased to bacterial counts of 7.2–7.9 log (CFU/g), except sugar kelp samples blanched in potable water, which had lower levels of 3.3–5.7 log (CFU/g). After seven days, presumptive *Pseudomonas* spp. levels in the four other treatments rose to 5–6.3 log (CFU/g) (Wirenfeldt et al. 2022). The initial load, microbial activity, and pretreatments influenced the quality and shelf life of seaweed, which need to be properly monitored during processing.

### 5.5 Packaging Technique

Throughout the supply chain, packaging is essential to extending the shelf life of food items. Modern packaging has made it possible for a wide range of items to be available all year round in different regions of the world. Active packaging is one such innovation that keeps food fresher longer by halting deterioration. Typically, active packaging consists of emitters and absorbers. The emitters release helpful compounds into the package to preserve the food, while the absorbers remove undesirable elements that cause food to degrade from the product and its surroundings (Kumar et al. 2018). A method called modified atmosphere packaging (MAP) is used to extend the shelf life of foods that are fresh or lightly processed. This technique suppresses the air surrounding the food in the packaging and replaces it with a gas (or combination of gases) of a different composition. The product type, packing materials, and storage temperature all affect the gas composition. MAP makes it possible to extend the products' original qualities (Zhang et al. 2016).

To examine the effect of packaging on seaweed quality during storage, a 15‐day study was carried out, in which both seaweeds remained within food‐safety limits, with MAP and Vacuum (VAC) conditions at 6°C ± 2°C, maintaining odor quality more effectively than the control (CTRL), especially for *Ulva lactuca* (Chlorophyta). In *Porphyra umbilicalis* (Rhodophyta), MAP and VAC reduced microbial counts over time, whereas CTRL increased, reaching 5.477 log CFU/g by day 6. VOC (volatile compound) profiles were also better preserved under MAP and VAC. *P. umbilicalis* decreased from 41 VOCs in fresh samples to only 8 in CTRL, but retained 12 and 8 under VAC and MAP, respectively. *U. lactuca* dropped from 60 VOCs to 24 in CTRL but remained higher in VAC (21) and MAP (24). Overall, MAP and VAC provided markedly better microbial and VOC stability than CTRL and performed almost equally well for both seaweeds (Moreira‐Leite et al. 2023). Here, it is represented that the methods that restrict oxygen can assure better quality than any kind of conventional system.

### 5.6 Temperature of the Storage Environment

At atmospheric pressure, microbial growth can take place at temperatures ranging from roughly −8°C to 100°C. Thermophiles, which have an optimum temperature of 55°C and a range of 45°C–70°C; mesophiles, which have an optimum temperature of 35°C and a range of 10°C–45°C; and psychrophiles, which have an optimum temperature of 15°C and a range of −5°C to 20°C. In foods exposed to temperatures above and below the minimum and maximum limits of growth, microbial cells die relatively slowly at lower temperatures and quickly at higher ones (Ashagrie and Abate 2012).

There is limited information regarding the storage conditions of seaweed. Seaweed quality has traditionally been maintained by regulating the storage temperature. *Ulva lactuca* is quite sensitive to variations in temperature. It deteriorates in a matter of days when kept at 16°C, exhibiting increased microbial activity. At 4°C, the greatest increase occurred on the sixth day, and at 16°C, it increased from the second day, reaching final levels of 5.2 × 10^8^ and 6.6 × 10^8^ microbial cells mL^−1^, respectively, and color degradation, such as loss of greenness, was also noticed. The loss of brightness occurred progressively from the first days of storage onwards, being more intense in the samples stored at 16°C (Sánchez‐García et al. 2021). Compared to other seaweeds that might have stronger structures or lower moisture content, this sensitivity results in a much shorter shelf life in tropical climates with higher room temperatures (Jasmadi et al. 2023; Moreira‐Leite et al. 2023). Thus, low‐temperature storage, like refrigeration, can be effective in preserving temperature‐sensitive seaweeds such as *U. lactuca*, slowing microbial growth.

### 5.7 Storage Condition (Relative Humidity)

Water activity in food and the development of microbes on food surfaces both depend on the relative humidity (RH) of the storage environment. In essence, relative humidity is a measurement of the gas phase's water activity. Food items with low water activity will absorb moisture until equilibrium is reached when they are kept in an atmosphere with a high relative humidity. Water will move from the gas phase to the food. In a similar way, foods with a high water activity lose moisture when exposed to low relative humidity (Awulachew 2021). The highest‐grade seaweed is produced by drying it at 60°C with a relative humidity of 10% (Fudholi et al. 2011). When the relative humidity of the drying air increased in the range of 20%–80%, blanched seaweeds linearly acquired moisture, leading to a significant degree of hysteresis between the sorption and desorption behavior (Tolstorebrov et al. 2024).

A previous study showed some findings related to humidity, where higher relative humidity impacted the color quality of stored seaweed. When comparing the lightness before and after incubation in 48% RH and 61% RH, the *L*‐value of thylakoid powders was significantly lower (indicating a darker color). At low relative humidity (10% RH and 32% RH), there was no significant change for spray‐dried powders. However, at higher relative humidity, the chlorophyll content dramatically dropped from 68 mg/g before incubation to 42 mg/g (−38%) and 32 mg/g (−52%) after incubation in 49% RH and 61% RH (Östbring et al. 2020). This suggests that controlling environmental humidity, especially for dried seaweed storage, is crucial to prevent further moisture absorption and pigment degradation.

The factors responsible for the quality and shelf life of seaweeds are found to be closely interrelated. Simultaneously controlling both environmental and packaging‐related conditions is necessary rather than regulating individual factors independently.

## 6 Shelf Life of Seaweed

Different studies found that the shelf life of seaweed is influenced by variation in species, form, treatment, packaging type, and storage temperature. Where refrigeration of seaweed alone generally exhibits a shorter shelf life than blanching, salting, freezing, and High‐pressure processing (HPP). For instance, freshly cleaned or mildly heated sugar kelp kept at 2.8°C was found to have a shelf life of 7–9 days, which is similar to previous findings on red, green, and brown seaweeds when stored in a refrigerator between 2°C and 7°C (Table 3). According to Nayyar (2016) and Nayyar and Skonberg (2019), seaweeds stored in refrigerated conditions have sensory shelf‐lives ranging from 3 to 14 days, depending on the species and washing method, supporting the fact of lower effectiveness of the refrigeration system than others. The negative impact of elevated storage temperature was also apparent in thawed and chilled *Undaria pinnatifida* (wakame) that had been kept at 10°C with a shelf life of 2–3 days (Choi et al. 2012), and sugar kelp held at the same temperature had a shelf life of 3.7 days (Wirenfeldt et al. 2022). On the other hand, López‐Pérez et al. (2020) reported that raw, untreated, brown macroalga *Laminaria ochroleuca* has a sensory shelf life of < 60 days when stored at 5°C, though the microbial concentration rose from starting values of 5 log (CFU/g) to 8 log (CFU/g) during the first 40 days of storage. Thus, this longer shelf life can be linked to species‐specific concepts or variations in sensory evaluation criteria among studies.

**TABLE 3:** Shelf life of seaweeds under different packaging and storage conditions.

Species	Form	Treatment	Packaging type	Storage temperature (°C)	Shelf life (days)	Initial microbial load (log CFU/g)	Final microbial load (log CFU/g)	References
*Ulva lactuca, Undaria pinnatifida*	Whole seaweed	HPP treated	Plastic sealed	4	180	4.24–6.14	7	Del Olmo et al. (2020)
*Ulva rigida*	Whole seaweed	Washed	Plastic sealed	4	10	7.93	8.72	Sánchez‐García et al. (2021)
*Ulva rigida*	Whole seaweed	Washed	Plastic sealed	16	6	7.93	8.82	Sánchez‐García et al. (2021)
*Porphyra umbilicalis*	Whole seaweed	Washed	Plastic sealed	6	6	4.775	> 5.477	Moreira‐Leite et al. (2023)
*Porphyra umbilicalis*	Whole seaweed	Washed	MAP	6	≥ 15	4.775	< LOD	Moreira‐Leite et al. (2023)
*Porphyra umbilicalis*	Whole seaweed	Washed	Vacuum	6	≥ 15	4.775	< LOD	Moreira‐Leite et al. (2023)
*Ulva lactuca*	Whole seaweed	Washed	Plastic sealed	6	15	3.335	3.6–3.8	Moreira‐Leite et al. (2023)
*Ulva lactuca*	Whole seaweed	Washed	MAP	6	15	3.176	3.8–4.1	Moreira‐Leite et al. (2023)
*Ulva lactuca*	Whole seaweed	Washed	Vacuum	6	15	3.176	3.699 (day 15)	Moreira‐Leite et al. (2023)
*Gracilaria* sp.	Nori	—	Aluminum foil sealed	25	89	—	—	Liviawaty et al. (2021)
*Laminaria* sp.	Gel granules	—	Vacuum‐sealed	4	> 20	≤ 4.48	≤ 4.48	Chen et al. (2024)
*Laminaria* sp.	Gel granules	—	Vacuum‐sealed	25	16	≤ 4.48	4.62	Chen et al. (2024)
*Saccharina latissima*	Whole seaweed	Untreated	Plastic sealed	2.8	7–9	4.0–4.5	7.2–7.9	Wirenfeldt et al. (2022)
*Saccharina latissima*	Whole seaweed	Blanched	Plastic sealed	2.8	7–9	0.9–1.8	3.3–5.7	Wirenfeldt et al. (2022)
*Saccharina latissima*	Whole seaweed	Blanched	Plastic sealed	2.8	7–9	0–2.0	7.2–7.9	Wirenfeldt et al. (2022)
*Laminaria ochroleuca*	Whole seaweed	Untreated	Sealed	4	60	5.42	8.47	Del Olmo et al. (2019)
*Laminaria ochroleuca*	Whole seaweed	(HPP (400 MPa, 5 min))	Sealed	4	180	2.53	< 3	Del Olmo et al. (2019)
*Laminaria ochroleuca*	Whole seaweed	(HPP (600 Mpa, 5 min))	Sealed	4	180	3.62	< 3	Del Olmo et al. (2019)
*Laminaria ochroleuca*	Whole seaweed	Salted	Sealed	4	180	5.42	3.94	Del Olmo et al. (2019)
*Laminaria ochroleuca*	Whole seaweed	Frozen	Sealed	−24	180	5.42	2.75	Del Olmo et al. (2019)
*Alaria esculenta*	Whole seaweed	Salted	Sealed	5	90	—	< 3.5	Perry et al. (2019)
*Palmaria palmata*	Whole seaweed	Blanched	Unpacked	1.7	11	—	—	Nayyar (2016)
*Gracilaria*	Whole seaweed	Blanched	Unpacked	7.2	10	—	—	Nayyar (2016)
*Saccharina latissima*	Whole seaweed	Blanched	Unpacked	1.1	12	—	—	Nayyar (2016)

Furthermore, several reviewed studies also indicate that combined preservation techniques are beneficial in increasing shelf life. For example, winged kelp, which had been lightly salted (*a*_*p*_ of 0.96, 30–50 g salt kg^−1^) and kept at 5°C, showed a 6‐week shelf life, challenging the widespread belief that kelp is a very perishable food item (Perry et al. 2019; López‐Pérez et al. 2020). Additionally, dry‐salted fresh *Alaria esculenta* at 0, 30, 50, 180, and 200 g/kg was kept for up to 90 days at 5°C. Across all treatments, microbial counts stayed constant (< 3.5 log CFU/g), and texture did not alter. Over time, the color changed from yellow to green as the proportion of salt increased. According to sensory evaluation, the 180 g/kg salt therapy was preferred. All things considered, *Alaria esculenta* was successfully preserved by dry salting for 90 days while being chilled (Perry et al. 2019). These observations demonstrate the efficiency of salting in increasing shelf life and maintaining microbial stability under lower‐temperature storage.

## 7 Common Preservation Techniques of Seaweed

Common techniques of seaweed products preservation include salting, canning, chilling, freezing, fermentation, super‐chilling, drying, smoking, frying, pickling/marinating, and pasteurization (Rabiepour et al. 2024). Finding effective preservation techniques that can maintain the quality of seaweed from the harvest stage to large‐scale processing or final use is crucial. Dehydration is considered one of the most common methods for the preservation of seaweeds (Albers et al. 2021). Moreover, highly used methods for seaweed preservation include drying and salting, both based on water activity (*a*_*w*_) reduction (Del Olmo et al. 2020).

### 7.1 Drying of Seaweed (Traditional and Modern)

High chemical and microbiological stability are attained by drying; however, unwanted microbial contaminants, including potentially harmful species, do not get removed completely (Del Olmo et al. 2018). Essential amino acids and carbohydrates of *Saccharina latissima* (Phaeophyceae) were effectively maintained when drying conditions were suitable (Stévant et al. 2018). Drying, however, reduced some of its medicinal chemicals (Gupta et al. 2011), and rehydrating the dried seaweeds involved leaching of the beneficial compounds (Cox et al. 2012). Volatile organic compounds (VOCs) may be lost during drying and enzymatic reactions, and auto‐oxidation may cause bad tastes to develop (Zhu, Healy, et al. 2022).

#### 7.1.1 Sun Drying

Sun drying is a slower and less efficient method of drying as it depends only on environmental factors (Table 4); the air temperature stays lower, and the humidity stays greater, particularly in windy or damp conditions. Consequently, it was found that the drying time was longer, and the seaweed ended up with a larger water content (17.6%) than it had with solar drying. Moreover, sun drying had an effectiveness of 9.1%, which was marginally less than oven drying (Suherman et al. 2018). However, there is a greater chance of being contaminated by dust, dirt, and other pollutants when one is directly exposed to the open environment. Sun drying is more advantageous than solar drying, while being the most affordable and accessible method (Fudholi et al. 2014). The process of sun‐drying (4 days) of *Sargassum oligocystum* (Phaeophyceae) had the largest ash level by far (32.83% DW). Sun‐dried samples had the greatest levels of saturated fatty acids (SFA) (52.896%), followed by samples that were oven‐dried (48.808%) for 36 h at 60°C and freeze‐dried (43.119%) for 48 h at 80°C. But when exposed to sunlight, polyunsaturated fatty acids (PUFA) were considerably lower (16.393%). Moreover, phosphorus content was also significantly higher in sun‐dried samples (0.15%) (Ullah et al. 2025). The analysis revealed that mineral retention is prominent here, but loss of PUFA may be higher due to oxidation in long time direct exposure.

**TABLE 4:** Effects of different drying methods on nutrient retention of seaweed.

Drying method	Key parameter	Nutrient retention	Advantages	Limitations	References
Sun drying	4 days under direct sunlight	Lower retention, Reduction of protein, amino acids, fatty acids, and antioxidants compared to oven and freeze‐drying	Low‐cost and minimal equipment are needed	Weather‐dependent, long drying time, Risk of contamination, nutrient loss	Ullah et al. (2025)
Oven drying	80°C for 24 h	Nutrient loss but higher amino acid content in *Fucus* sp. (~17.3 g kg^−1^, DM) compared to 40°C (~15.1 g kg^−1^, DM)	Simple, widely available, and controlled temperature	High temperature reduces fatty acids and polyphenols	Dhakal et al. (2024)
	60°C for 36 h	Moderate retention: amino acids (81.263%) are higher than sun drying but lower than freeze drying, Reduction of fatty acids, minerals, phenolics, flavonoids, Both phenolic and flavonoid content in *Padina* sp. increase with oven drying (60°C > 80°C > 100°C)	Simple, widely available, Controlled process	Higher temperature reduces fatty acids and antioxidants, Higher energy use	Ullah et al. (2025), Mingu et al. (2024)
Microwave drying	385 W, 15 min	Preservation of certain chemicals, Potentially beneficial compared to oven drying in some cases, Antioxidant activity and nutrient levels are species‐dependent *Laminaria digitata* and *Halidrys siliquosa* showed higher PUFA contents	Rapid drying reduces the risk of contamination and oxidation, Energy‐efficient	Can cause uneven heating, Nutrient retention varies with species and power level	Badmus et al. (2019)
Convective drying	50°C/60°C	50°C: best balance (highest diffusion coefficient, good sensory quality), 60°C: significant loss of flavor & metabolites, preserved some fatty acids	Efficient, cost‐effective, industrially scalable, 50°C gives a good balance between efficiency and quality	Higher temperature (60°C) degrades the sensory and metabolite profile, Less effective in preserving phenolics/flavonoids than freeze‐drying	Yin et al. (2025)
Freeze drying	–50°C for 72 h	Preservation of fatty acid, stable protein factor, Polyphenol: slightly affected,	Nutrient preservation, Minimal damage,	Costly and time‐consuming, Energy intensive,	Dhakal et al. (2024)
	−80°C for 48 h	Best retention of nutrients: amino acids (86.544%), MUFA, PUFA, Phenolics (62.994 mg GAE/g), flavonoids (46.151 mg QE/g), antioxidants in *Sargassum*	Excellent nutrient and antioxidant preservation, Minimal thermal damage	Very expensive and time‐consuming, Energy‐intensive	Ullah et al. (2025)

A similar trend of lower efficiency of sun drying was also reported by Khan et al. (2025), comparing sun (8 h) and solar drying (8 h), they discovered that, with a sample size of 100 g, solar drying resulted in a greater loss of moisture, which led to much lower final weights for *Sargassum polycystum* (43.8 g) and *Gongolaria barbata* (formerly *Cystoseira barbata* ) (Phaeophyceae) (40.1 g) than open sun drying (55.81 and 52.9 g, respectively). Seaweed dried more quickly in the solar dryer, with *Sargassum polycystum* and *Gongolaria barbata* achieving 7.03 and 7.5 g/h, respectively, as opposed to 5.52 and 5.89 g/h for open sun drying. Furthermore, *Sargassum polycystum* and *Gongolaria barbata* achieved 9.52% and 10.13% drying efficiency, respectively, using the solar dryer, compared to 7.4% and 7.9% for open sun drying. Here, less efficient drying is noticed for sun drying, which can lead to further loss of nutritional compounds. On the whole, sun drying is cost‐effective and easily accessible, but the contamination risk is reported to be higher. Hence, its use should be lower when safety and quality are the main concerns.

#### 7.1.2 Oven Drying

When seaweed is dried in an oven, the water removal pattern is slower and less effective than when it is dried in the sun. It was discovered that the ultimate moisture content stayed at 21.82%, which was explained by the drying chamber's uneven heat distribution, which hindered the evaporation of moisture. Even while oven drying offers weather‐independent controlled conditions, its drying effectiveness was the lowest of all methods. Although it provided more energy, a large portion of it was used ineffectively to remove water. Oven drying is worse in terms of efficiency and drying rate than tray‐type drying systems, which have warm air come into closer contact with the material (Suherman et al. 2018). Furthermore, oven drying is less efficient because it lowers the quality of the product in terms of texture and color. Oven‐drying has been shown to intensify the yellow tonality in the green microalgae *Arthrospira* sp. The authors hypothesize that this is due to the breakdown of chlorophylls (Larrosa et al. 2017).

In another study, the a* parameter for *Fucus vesiculosus* (Phaeophyceae) did not significantly (1.12 ± 0.3 to 1.46 ± 0.50) alter after oven drying at 60°C when compared to control samples. While yellow tonalities and luminosity increased (b* values of 1.43 and 4.68 and L* of 2.15 and 4.41, for temperatures of 40°C and 60°C, respectively). With a* values ranging from 4.86 ± 0.47 to 10.09 ± 0.71, oven drying *Gracilaria* sp. (Rhodophyta) and *Ulva rigida* (Chlorophyta) had a greater effect on the a* coordinate than on the b* coordinate. The IC50 values for the control and oven‐dried samples ranged from 0.06 to 0.09 mg/mL, respectively. The water retention curve (WRC) of freeze‐dried and oven‐dried samples did not differ significantly overall, which may indicate that heat drying conditions have an undetectable effect on the cell wall structure of *Ulva rigida* , *Gracilaria* sp., and *Fucus vesiculosus* (Silva et al. 2019). The differential color changes observed among green, red, and brown seaweeds likely result from variations in pigment composition.

#### 7.1.3 Microwave Drying

Moisture removal is quick and effective with microwave drying. In contrast to the sluggish transmission of heat through frozen layers in freeze‐drying, microwave energy enters the product directly, allowing water molecules to be excited and accelerating evaporation at lower pressures (Scaman et al. 2014). As a result, sea lettuce and bladder wrack dried in 3–4 h as opposed to 24 h for freeze‐drying. Beta‐carotene and phenolic content were maintained at levels equivalent to or better than freeze‐drying, and higher concentrations of nutritionally significant chemicals, such as free aspartic acid, fucoxanthin, and total glutamic acid, were retained in bladder wrack using this approach. According to a sensory study, microwave‐vacuum drying maintained delicate flavors associated with seaweed, like umami and hay, although the overall product quality fell somewhere between convection and freeze‐drying (Wirenfeldt 2023).

Microwave‐vacuum drying shows promise as a substitute for freeze‐drying, preserving essential bioactive components while cutting drying time and balancing efficiency and product quality. Consistently low total antioxidant capacity (TAC) was observed for microwave‐dried *Fucus spiralis* (Phaeophyceae). *Halidrys siliquosa* (Phaeophyceae) showed higher total phenolic content (TPC) than the rest of the species, with considerably higher levels for microwave‐dried and freeze‐dried batches compared to oven‐dried. The protein content of *Fucus spiralis* and *Pelvetia canaliculata* (Phaeophyceae) was also higher after low temperature oven drying (40°C, 48 h) when compared to high temperature oven drying (60°C, 48 h) and microwave treatments. All species tended to show the highest lipid content in microwave‐dried batches, except for *Laminaria digitata* (Phaeophyceae), where 40*°*C gave marginally higher results in the oven drying (Badmus et al. 2019). As changes in compounds are reported among species, drying conditions should be optimized based on seaweed species and parameters evaluated.

#### 7.1.4 Convective Drying

Convective drying, sometimes referred to as hot air drying, is a commonly used method that uses forced or natural convection to circulate warm air to remove moisture from seaweed. The fastest drying rate was achieved by applying forced air to both bladder wrack and sea lettuce at 52°C (Wirenfeldt 2023). Air temperature, airflow speed, and relative humidity all affect how well convective drying works because these factors produce a vapor pressure difference between the seaweed and the surrounding air, which makes it easier to remove moisture (Rahman 2020). Higher temperatures, however, have the potential to cause case hardening and degrade the product's chemical and sensory qualities. Convective drying results showed notable modifications to the physicochemical and sensory qualities. Compared to freeze‐drying and microwave‐vacuum drying, it created a curlier texture and increased the water activity (*a*_*w*_), water absorption (WA), and water holding capacity (WHC) of bladder wrack. Just free glutamic acid underwent a drop in chemical composition. Aspartic acid, fucoxanthin, and total glutamic acid either stayed the same or rose (Wirenfeldt 2023).

The influence of Convective drying (CD) on seaweed has been investigated and noticed that it altered the composition and quality of *Ulva* spp. The ash content of CD (70°C, air flow rate = 2.0 ms^−1^) samples was comparatively greater (19.65 g/100 g) than that of samples that were freeze‐dried (−50°C, 0.027 kPa, 68 h) and vacuum dried (70°C, 15 kPa). The fiber content was 6.28–6.98 g/100 g, while the (insoluble dietary fiber/soluble dietary fiber) IDF/SDF ratio was lower (1.27). Furthermore, under CD, the total phenolic content (TPC) decreased by roughly 37% when compared to freeze‐dried samples (Uribe et al. 2019). This was explained by the high drying temperature and tissue dehydration, which degrade phenolics (Gupta et al. 2011). The reports as a whole exhibit that, for convective drying to produce the desired results, managing the drying temperature is crucial for maintaining product quality.

#### 7.1.5 Freeze‐Drying

Freeze drying, sometimes referred to as lyophilization, is a sophisticated preservation method that preserves the product's structure, flavor, and nutritional qualities by sublimating materials under low‐temperature and vacuum settings (Albers et al. 2021). This process works especially well for keeping heat‐sensitive nutrients, bioactive substances, and pigment‐like phycocyanins and fucoidans, which are frequently destroyed by traditional drying methods (El‐Beltagi et al. 2022). Seaweed shelf life is increased by freeze‐drying, which also reduces microbiological growth and enzymatic reactions by lowering moisture content. It also reduces weight and volume, making storage and shipping easier (Alp and Bulantekin 2021). Numerous industries have used freeze‐dried seaweed, including agriculture and aquaculture (Kinley et al. 2020), pharmaceuticals and healthcare for wound dressings and drug delivery systems (Klojdova et al. 2023), nutraceuticals and dietary supplements (Lomartire et al. 2021), and culinary products like snacks, seasonings, and kelp powders (Coleman et al. 2023). By increasing energy efficiency and lowering greenhouse gas emissions, contemporary freeze‐drying methods further highlight environmental sustainability. Seaweed and other delicate materials can be preserved using freeze‐drying in a variety of ways that preserve their commercial, nutritional, and functional value (Ashworth et al. 2024).

Few studies showed species‐specific differences of the method, where total phenolic compounds (TPC) recovered from *Ulva rigida* and *Gracilaria* sp. were much lower than those extracted from *Fucus vesiculosus* (approximately 1 vs. 11 mg EGA/g seaweed, in samples stabilized by freeze‐drying) (Silva et al. 2019). *Sargassum oligocystum* that was freeze‐dried (−80°C, 48 h) maintained the highest levels of protein (9.98% DW), lipids (0.78%), and fiber (7.47%), as well as the noticeably highest moisture content (94.46% FW). The highest concentrations of sodium (0.71%), potassium (2.54%), calcium (1.87%), and magnesium (1.43%) were all retained by freeze drying. Those that were freeze‐dried had the greatest cumulative TAA content (86.544%), followed by those that were oven‐dried (60°C, 36 h) (81.263%) and sun‐dried (4 days) (75.779%). Sun drying produced the considerably lowest values for all parameters, whereas oven drying produced the significantly higher TPC (62.994 mg GAE/g), TFC (46.151 mg QE/g), and antioxidant activity (71.13% ABTS and 74.435% DPPH scavenging) (Ullah et al. 2025). It reflects that the effectiveness of freeze‐drying varies according to seaweed species and the evaluated quality parameters.

Among the evaluated drying techniques, the better efficiency of freeze‐drying preservation is mainly related to the low‐temperature dehydration, thus reducing thermal degradation of sensitive bioactive and nutritional compounds. On the other hand, microwave and convective drying are more efficient in industrial applications.

### 7.2 Salting

Salting is a widely employed preservation technique; however, certain salt‐tolerant microbial populations in salted seaweeds may persist during storage, indicating that salting alone may not always ensure microbiological safety (Del Olmo et al. 2018; Perry et al. 2019). Salted kombu's total polyphenol content and antioxidant capacity significantly decreased while it was refrigerated (Del Olmo et al. 2019), and phytochemicals may also be lost if it is desalted before consumption. The process of salting preservation involves lowering the water content to prevent the growth and development of microorganisms. The microbial cell membrane may be broken down by the osmotic qualities of high salt, and the hygroscopic properties may disrupt the activity of proteolytic enzymes and dissociated chloride ions. Since salting is not a one‐stop cure, further methods like boiling or desiccation are typically used. Additionally, salt is essential to the development of flavors, textures, and odor. Wet/dry and wet‐dry combination salting methods are offered, and a curing procedure is also used. The sodium chloride (NaCl) salt is either a solution or a crystal. The usage of nitrate and nitrite salts is part of the curing process (Indiarto et al. 2021). According to Wei et al. (2021), refrigeration preservation at a salinity of 10% was also effective in preserving several seaweed species, even when stored at room temperature.

In another study, brine‐salted kelp received more sensory acceptance than dry salting. The sodium concentration of the rinsed brined kelp (40% w/v NaCl), dry salted kelp (30% (w/w)), was 16.45 ± 0.65 g/kg (w.w.b.) and 12.9 ± 0.94 g/kg (w.w.b.), respectively. On the 9‐point hedonic scale, consumer approval of kelp salads appears to have been positively impacted by salt treatment according to the overall sensory assessment liking scores for the salad treatments (control, 5; dry salted, 6; and brined, 6.3). According to penalty analysis, the percentage of customers who thought the control salad samples were “too tender” may be connected to this decreased acceptance (Arya et al. 2024).

### 7.3 Freezing

Freezing to a temperature of below −25°C is a good way to prevent microbial development while storing, but the plant may become more susceptible to microbes after thawing due to the damage done to the cell structure during freezing and thawing (Table 5) (Del Olmo et al. 2019). To reduce the chance of microbial growth and to decrease drip loss, it is advised to freeze and thaw quickly (Løvdal et al. 2021). The effect is easily reflected in *Alaria esculenta* (−26°C), showing a 57% drip loss of the original sample wet weight. The drip loss of the *A. esculenta* raw material sample resulted in the loss of 60% of the ash content and 17% of the dry matter, excluding ash. The original raw material had a protein content of 8.0% ± 0.4% of DW, according to the sum of all amino acids, with 6.3% of that content lost to drip loss. Alanine was the primary amino acid that was lost to the drip loss samples during thawing. 62% of the carbohydrate content associated with the studied chemicals in the original raw material was lost to drip loss, according to the total concentration of these in the three fractions (Sund et al. 2024). There is surprisingly little literature available on the freezing of seaweeds, possibly due to the limited changes during long‐term frozen storage.

**TABLE 5:** Common preservation techniques of seaweed.

Method	Principle	Advantages	Disadvantages	Typical applications	Species	Example	References
Drying (sun)	Removal of moisture from a material by evaporation, applying heat, air circulation, or a combination of both	Low cost, Easy to practice, Extended shelf life, Retains higher phenol‐(1.59 mg GAE/g) in dried *Kappaphycus* and higher flavonoid in *padina*‐(0.95 QUE/g)	Improper process may cause shrinkage, Loss of some nutrients, Discoloration and texture loss	Food, nutraceutical, healthcare, agriculture, aquaculture feed industry	*Laminaria* sp., *Ulva* sp., *Enteromorpha* sp., *Sargassum* sp., *Gracilaria* sp., *Kappaphycus*	Salmon, tilapia, and cattle feed, seaweed snacks, seasoning, powder, or flakes	Rajauria et al. (2015), Ancajas et al. (2023), Nowak and Jakubczyk (2020), Mingu et al. (2024)
Salting	Application of dry salt (0–200 g/kg) or brine to decrease water activity (*a*_*w*_), causing hyperosmotic shock, altered microbial metabolism, and toxic effects of chloride ions	Inhibits spoilage and pathogenic microorganisms, Extends the shelf life of kelp up to 90 days when combined with freezing, Used as a stand‐alone or pre‐treatment for drying/fermentation, Low cost and traditional method	High salt content may reduce nutritional and sensory quality, Requires rinsing before consumption, Empirical methods may cause inconsistent quality, May not fully align with modern low‐sodium health trends	Food industry	*Undaria pinnatifida*	Salted wakame in South Korea, France (Brittany), Spain (Galicia)	Perry et al. (2019)
Freezing	Conversion of water into ice crystals at subzero temperatures, slowing microbial growth and enzymatic/biochemical reactions	Elimination of detectable aerobes in untreated samples at 11 days Slows enzymatic degradation, Maintains texture, color, and nutrients better than drying, Large‐scale technology	Drip loss upon thawing (27%–50% WW), Loss of bioactive and nutritional compounds, Ice crystals damage tissue structure and texture, Energy‐intensive due to the cold chain	Food and feed industry	*Saccharina latissima*, *Gracilaria* sp., *Ulva* sp.	Frozen kelp, frozen seaweed puree	Stévant (2019), Stévant et al. (2024)
Fermentation	Conversion of polysaccharides and sugars into organic acids (lactic, acetic, etc.) by microorganisms (LAB, yeast)	Enhances nutritional value (prebiotic and probiotic effects), Improves flavor and texture, Provides bioactive, antioxidant, and antimicrobial properties, Reduces Cd and Hg in *Saccharina latissima*	Limited scientific studies on seaweed fermentation, Risk of inconsistent quality (depends on microbial strain and process), Requires controlled conditions to ensure safety	Functional foods, beverages, animal feed, fertilizers, nutraceuticals, and food coating	*Undaria pinnatifida*, *Gracilaria fisheri*, *Ulva lactuca* , *Caulerpa* spp., *Sargassum fulvellum, Saccharina latissima*	Fermented wakame as aquaculture feed and fermented beverage from *Gracilaria fisheri*	Martelli et al. (2020), Monteiro et al. (2021), Bruhn et al. (2019)
Blanching	Heating seaweed in hot water or steam for a short time, altering chemical and mineral composition, reducing compounds (e.g., iodine (I), arsenic (As), sodium (Na), potassium (K))	Reduces toxic mineral content, Improves nutritional profile (70°C for 3 min), Improves digestibility: IVOMD (60.7 → 63.0%), Reduces methane when used as feed (3%–5%) in *Sargassum horneri*	Causes loss of sugars, proteins, and water‐soluble minerals, Excessive blanching may reduce digestibility, High supplementation levels (> 10%) can cause feed refusal in livestock	Food pre‐treatment and the livestock feed industry	*Ascophyllum nodosum* , *Sargassum* sp. (*S. horneri*)	Blanched *Sargassum horneri*, dairy cattle feed with *Ascophyllum nodosum* and *Asparagopsis* supplementation, reducing methane by 26.4%	Wardani et al. (2024)

Pre‐treatments (PTs) and freezing had a major impact on *Saccharina latissima* quality and chemical makeup. Blanching (60°C, 2 min) and steaming (95°C, 15 min) resulted in greener samples, while salting (2:3 = salt: kelp for 1 h) raised the dry matter content before freezing (−20°C ± 0.9°C). In comparison to the 15%–24% seen in untreated samples, drip loss in steamed samples varied from 11% to 16%. WSP (*water* sorption) retention was highest in salted samples (0.61 ± 0.07) and lowest in blanched samples (0.36 ± 0.04), while iodine retention was lowest in blanched samples (0.07 ± 0.01). Untreated biomass had a total monosaccharide and uronic acid (TMUA) content of 38.5 ± 8.1 g/100 g dry weight (DW), primarily mannitol (31.5% of TMUA) and glucose (10.7%) (Stévant et al. 2024). Hence, evaluations revealed that short‐term pretreatments should be taken into consideration since they have a significant influence on nutrient component retention during freezing by reducing the impact of drip loss.

### 7.4 Fermentation

One effective way to maintain the safety and integrity of a very perishable and unstable harvested wet biomass is through seaweed fermentation (Monteiro et al. 2021). *Pyropia yezoensis* (nori), a protein‐rich red seaweed, was successfully used to make a high‐salt sauce by the fermentation process of halophilic lactic acid bacteria (LAB) (Uchida et al. 2017). Fermentation, which was primarily created to stabilize perishable agricultural items (Terefe 2016), might also be a better processing technique for seaweed species that are more susceptible to physical treatments like freezing and heat. Since *Lactobacillus acidophilus* and *Lactobacillus plantarum* can ferment galactose, the primary sugar found in red seaweeds, preparations of *Gracilaria* sp., *Sargassum siliquosum*, and *Ulva lactuca* acid and cellulase hydrolysate were tested as substrates for lactic acid production (Lin et al. 2020). LAB fermentation demonstrated efficacy when seaweed grown was combined with sauerkraut at a ratio of up to 1:1. This resulted in a suitably low pH, which maintained acceptable microbiological and sensory quality for up to 60 days after inoculation (Skonberg et al. 2021). Although this is above the limit set at 4.3 regarding the growth of *Bacillus cereus*, no colonies with the morphology of *Bacillus cereus* were observed after 40 h of heat treatment (95°C for 15 min) followed by fermentation using a commercial *Lactobacillus plantarum* starter culture. This resulted in a drop in pH and stabilization at pH 4.5 (Bruhn et al. 2019).

A study was conducted on fresh *Saccharina latissima* seaweed based on different concentrations of lactic acid (LA) (3, 6, and 9 g lactic acid per kg (FW)) and one concentration of citric acid (3 g citric acid per kg FW). The findings showed that the preserved samples had a level of K that was reduced by about 50%. Furthermore, it was discovered to be 58.0 ± 0.8 g kg^−1^ DW, and it rose as the concentration of LA decreased. Additionally, moist weight or total biomass loss was the highest, at 30.6% ± 1.2%. Salinity and sourness were greatly affected by the treatments, with sourness scores ranging from 2.8 ± 1.0 to 4.9 ± 2.3 and saltiness scores ranging from 6.0 ± 1.3 to 7.2 ± 0.9 on the 9‐point scale (Krook et al. 2024), resulting in a flavor profile characterized by greater saltiness and lower sourness. Researchers discovered that the fermentation of the brown seaweed *Undaria pinnatifida* substrate produced varying amounts of ethanol and lactic acid. For example, the values in the blade ranged from 0.18 to 0.23 g 100 mL/L for lactic acid production and from 0.07 to 0.38 g 100 mL/L for ethanol synthesis (Uchida and Miyoshi 2013). It reflects that the identification and application of specific concentrations and substrates are important here for better findings.

### 7.5 Blanching

Seaweeds are blanched and boiled for a number of reasons, including to deactivate bacteria and natural enzymes that break down the substance. Boiling for a few minutes can reduce the unacceptably high iodine levels found in brown seaweeds by up to 94%. However, boiling reduces the incidence by destroying water‐soluble minerals and flavonoids (Ho and Redan 2021). It has been proposed that blanching before certain of these preservation techniques, such as drying and freezing, can slow down the rate at which products deteriorate (Del Rosario and Mateo 2019). Fucoxanthin, a dark pigment that suppresses the green color of chlorophyll in raw kelp, breaks down when blanched kelp exhibits a high intensity of greenness (Zhao et al. 2019). In comparison to the shorter blanching time of 1 min, the prolonged blanching time of 3 min at 100°C produced a lower green intensity. As the blanching duration increased, the stiffness of the kelp diminished, indicating that the polysaccharides in the kelp cell walls were broken down by heat. Given that final products are sold by weight, there is a chance that the higher moisture content will boost kelp processors' earnings. Because blanching improved the kelp's moisture content, lightness, and greenness—all of which improved sensory scores—it could help commercialize kelp products (Akomea‐Frempong et al. 2021).

Blanching improved the quality of *Sargassum horneri* by reducing some excessive minerals, where sodium (Na), potassium (K), and arsenic (As) decreased from 29.4 to 15.5, 171.7 to 50.4, and 3.16 to 1.13 mg/g, respectively, with increasing temperature (80°C). A linear drop in crude protein concentration (15.8% vs. 15.5% vs. 15.0%) was seen with an increase in blanching duration (1 vs. 2 vs. 3 min). The high temperature (80°C) with 1 min of blanching and the medium temperature (70°C) with 2 min had the largest crude protein content. Digestibility improved slightly with in vitro organic matter digestibility (IVOMD) and in vitro *dry matter digestibility* (IVDMD) rising from 51.0% to 54.0% at higher temperatures. Additionally, the optimal blanching condition is recognized as 70°C for 3 min, which balances nutrient retention and mineral reduction. Improper blanching causes loss of soluble nutrients: DM (Dry Matter, 10.1 → 7.02%), CA (Crude Ash, 31.9 → 15.3%), and CP (Crude Protein) decrease with time (15.8 → 15.0%) (Wardani et al. 2024). This indicates the necessity of optimizing temperature and duration to avoid leaching.

The suitability of seaweed preservation methods is noted to be highly dependent on the processing objective, since the methods have different effects on storage stability, microbial control, sensory quality, and product functionality. This specifies the necessity of selecting preservation techniques in accordance with the intended industrial and quality outcomes.

## 8 Advanced Preservation Techniques of Seaweed

**FIGURE 3:**
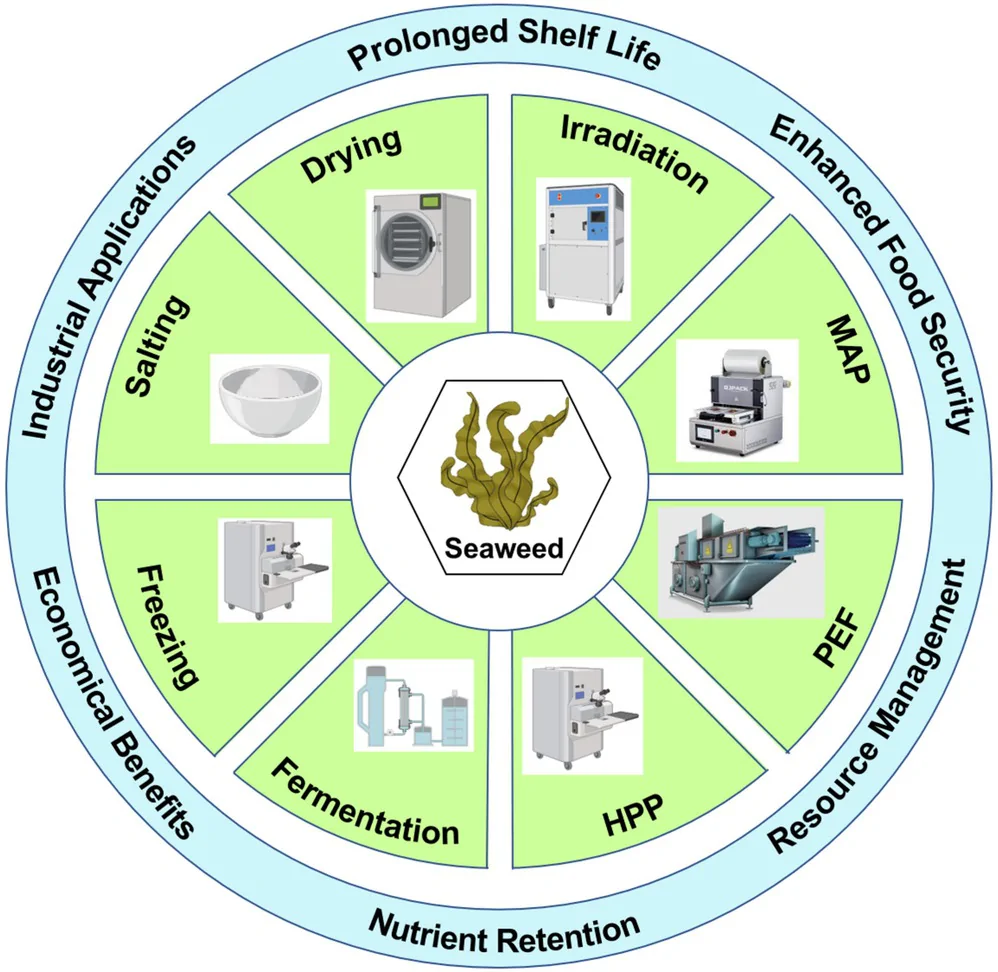
Seaweed preservation techniques used globally. The idea of the diagram was taken from ChatGPT, which was later modified by the authors in Microsoft PowerPoint.

Advanced preservation techniques for seaweed products, including ultrasound, pulsed electric field (PEF), irradiation, high hydrostatic pressure (HHP), microfluidization, natural preservatives, advanced packaging methods (MAP, active, and vacuum packaging), nanotechnology, and other emerging technologies, can effectively extend shelf life while improving the sensory properties, quality, and safety of the products. However, improving the weaknesses of traditional methods by using modern techniques can improve and maintain the nutritional value of the product (Rabiepour et al. 2024) (Figure 3).

### 8.1 High‐Pressure Processing

This is a modern method where the physiology and biochemistry of certain seaweeds were examined in relation to high‐pressure processing (Table 6). However, the pressure levels that were applied (20–100 MPa) were too low to have an impact on microorganisms. Microbial growth is at least partially responsible for the shelf life of seaweeds, and HPP can regulate this growth at higher pressures. According to Del Olmo et al. (2018), pressure levels of 400–600 MPa were used to improve the microbiological quality and safety of kombu ( *Laminaria ochroleuca* ). This had a positive impact on the shelf life, which reached 180 days at 4°C. Additionally, following refrigeration, the odor features and high odor acceptance scores of kombu treated with HPP were preserved (López‐Pérez et al. 2020). Some of the properties of plant foods are known to be changed by HPP (Sánchez‐Moreno et al. 2009). It is likely to have varying effects on the traits of different seaweed species. Various edible seaweeds may require various HPP treatment parameters since seaweed microbiota varies significantly between species (Del Olmo et al. 2019).

**TABLE 6:** Advanced preservation techniques of seaweed.

Method	Principle	Advantages	Disadvantages	Typical applications	Species	Example	References
High‐pressure processing (HPP)	Non‐thermal preservation technique applying very high pressure (400–600 Mpa) to inactivate microorganisms and enzymes without heat damage	Extends shelf life (up to 180 days at 4°C for kombu), Maintains odor, color, texture, and antioxidant properties, Effective for microbial reduction, Retains fresh‐like quality	Requires high‐cost equipment, Low pressure (20–100 MPa) is ineffective against microbes, Different seaweeds respond differently, requiring optimized conditions	Food industry	*Laminaria ochroleuca* , *Chondrus crispus* , *Codium fragile* , *Himanthalia elongata* , *Ulva lactuca* , *Undaria pinnatifida*	Processed kombu	Del Olmo et al. (2020)
Pulsed electric field (PEF)	Application of short, high‐voltage electric pulses (10–80 kV/cm), which induce electroporation, increasing cell membrane permeability	Non‐thermal, non‐chemical, energy‐efficient, Enables the extraction of proteins and bioactive compounds, Disinfection potential, Parameter flexibility (voltage, pulses, duration, frequency)	Rigid seaweed cell wall limits the release of molecules, Requires optimization of parameters for each biomass type, Enzymatic pretreatment is needed for effective protein recovery	Cosmetic industry	*Ulva lactuca, Alaria esculenta, Palmaria palmata*	Seaweed extract	Castejón et al. (2021), Steinbruch et al. (2024)
Modified atmosphere packaging (MAP)	The surrounding environment of the product is replaced with a gas combination (CO_2_, N_2_, O_2_) that differs from the ambient condition	It inhibits the growth of microorganisms, Delays the synthesis of trimethylamine (TMA) and total volatile basic nitrogen (TVB‐N), Partially preserves good sensory qualities	Requires temperature control, Has additional expenses, Calls for specialized equipment	Food industry	*Ulva lactuca, Porphyra umbilicalis*	Seaweed laver	Esteves et al. (2021), Moreira‐Leite et al. (2023)
Irradiation	Preservation technique using ionizing gamma radiation (3–7 kGy) to inactivate microorganisms and improve hygienic quality without heat involvement	Improves microbial safety (eliminates total bacteria, coliforms), Maintains water content, pH, *a*_*w*_, protein, and ash, Increases viscosity, desirable for food texture, Environmentally friendly and residue‐free	Requires controlled dose and storage conditions to preserve bioactive components, High doses may affect antioxidant activity	Food industry	*Ecklonia cava*	Seaweed‐based irradiated functional food ingredients (carrageenan and alginate)	Yang et al. (2020), Kadir et al. (2023)

High‐pressure processing (HPP) has been effectively applied to various seaweed species to enhance extraction efficiency and extend shelf life. For instance, *Sargassum muticum* subjected to 300 MPa for 5–5.5 min achieved a polysaccharide extraction yield of 32%–40.4% (Rodrigues et al. 2017), while *Fucus vesiculosus* processed at 600 MPa yielded 23.7% protein. Similarly, *Alaria esculenta* , *Palmaria palmata* , and *Chondrus crispus* treated at 400–600 MPa for 5 min produced yields of 15%, 14.9%, and 16.1%, respectively. In the case of *Laminaria ochroleuca* (Kombu), storage at 4°C after HPP (600 MPa) treatment resulted in a shelf‐life extension of up to 180 days, accompanied by a reduction in viable bacterial counts by 2.53–3.62 log CFU/g on day 1 (Del Olmo et al. 2019).

Likewise, edible seaweeds *Himanthalia elongata* (Phaeophyceae), *Codium fragile* (Chlorophyta), *Ulva lactuca*, *Chondrus crispus*, and *Undaria pinnatifida* (Phaeophyceae) were subjected to a processing method (high‐pressure processing at 400–600 MPa for 5 min), and then stored (refrigerated storage at 4°C for 180 days) to examine the effects of HPP on microbiota, shelf life, physicochemical properties, color, texture, antioxidant capacity, and enzymatic activity during refrigerated storage. In most species, HPP treatment at 600 MPa increased shelf life by up to 180 days, and viable counts ranged from 4.24 to 6.14 log CFU/g on day 1 while largely preserving nutritional qualities like antioxidant capacity and polyphenol content. HPP has been demonstrated to be an effective preservation method under refrigerated storage (Del Olmo et al. 2020) and aligns with preceding examples showing the effectiveness of high‐pressure processing in extending shelf life. These findings illustrate how both minimal processing and controlled cold storage can contribute to extending seaweed shelf life through these mechanisms.

### 8.2 Pulse Electric Field

One new nonthermal and energy‐efficient advanced food processing method is the pulsed electric field (PEF). When a product is positioned between two electrodes, PEF applies electric field pulses, often with high voltages (kV range) and brief durations (micro‐ or nanoseconds) (Geada et al. 2018). Applying electric pulses causes reversible or irreversible holes to form in cell membranes, a process known as electroporation or electro permeabilization. This allows solvents to diffuse quickly and improves intracellular compound mass transfer (Poojary et al. 2016). Pulsed electric energy has been employed in recent applications to extract food and agricultural products (Vorobiev and Lebovka 2016). PEF treatment makes it possible to reduce the extraction time, raise the extraction rate of bioactive substances like anthocyanins, carotenoids, or polyphenols, and generate extracts with higher purity while doing away with the need for organic solvents (Käferböck et al. 2020). Proteins, carbohydrates, lipids, and pigments like carotenoids, chlorophylls, or phycocyanins from microalgae and seaweeds are just a few of the valuable commodities that have been effectively extracted from various marine sources with PEF treatment (Castejón et al. 2021).

PEF can potentially reduce excessive iodine content and other undesirable elements; for instance, in *Saccharina latissima*, PEF processing (energy levels: 14.4 kJ/kg) resulted in a 40% decrease in iodine content. This suggests that the electroporation settings used enhanced the permeability of hydrophilic iodine species, particularly iodide, over the kelp's cell membrane. The PEF procedure resulted in a final mercury level of 0.02 mg/kg dry sample, a substantial 19% reduction when compared to positive control samples. Compared to the other treatments, the mean content of lead was significantly greater following the high PEF treatment. While the other values fall within the range of the other treatments (0.6–2.0 mg/kg dry sample), this was brought on by one high value (13 mg/kg dry sample) (Blikra et al. 2022). For these reasons, PEF is generally helpful to extract elements in a rapid extraction system and ensures product safety. But for effective results, the settings and processing parameters should be well controlled.

### 8.3 Modified Atmosphere Packaging

The latest technique, known as “modified atmosphere packaging” (MAP), increases the shelf life of fresh or barely processed foods. MAP enables the extension of the original properties of the product (Zhang et al. 2016). MAP was found to be an efficient way to restrict the respiration rate of minimally processed seaweeds, specifically sea‐lettuce ( *Ulva lactuca* ) and laver ( *Porphyra umbilicalis* ), keeping their color and texture throughout a 15‐day storage period at 6°C. Under modified and vacuum atmospheres, the treatment dramatically decreased the microbial load in *P. umbilicalis*, whereas *U. lactuca* maintained rather steady microbial levels with no distinguishable variations between treatments and the control (Moreira‐Leite et al. 2023).

A study found that the microbial counts in *Ulva lactuca* treated with MAP stored at 6°C ± 2°C varied the most over the course of 15 days of storage, ranging from 3.176 log CFU/g on day 0 to 4.124 log CFU/g on day 2. In terms of texture, there was typically no significant change between the control and MAP treatments. The study identified the following volatile compounds (VOCs): one containing nitrogen, four containing sulfur, four halogenated VOCs (two containing bromine, one containing chlorine, and one containing iodine), one epoxide, one benzothiazole, eight furans, three indans, one naphthol, four phenols, one terpene, and three terpenoids. For the majority of samples, with a few exceptions, the number of chemicals identified did not significantly change throughout the course of the storage days (Moreira‐Leite et al. 2023). Thus, MAP can efficiently retain sensory and volatile components, though microbial stability showed species and storage condition dependency.

### 8.4 Irradiation

The major issue with regionally produced food items, such as seaweed, is the high degree of microbial contamination. To get over these issues, irradiation technology can be used. Food‐borne pathogens can be eliminated by radiation (Ajibola 2020). According to Ashtari et al. (2019), radiation can prolong the quality of food and enhance its safety, including seaweed or other heat‐sensitive bioactive ingredients. It can also solve the issue of fruit quarantine. Numerous studies on the effects of moderate doses of radiation (< 10 kGy) on the quality of semi‐wet processed foods and postharvest food demonstrate that irradiation can increase shelf life, decrease the overall number of microorganisms, and eradicate harmful microbes (Akinloye et al. 2015).

In a study on seaweed‐based functional food ingredients such as carrageenan and alginate, samples were irradiated at doses of 3, 5, and 7 kGy, using 0 kGy as the control. The results demonstrated that the water content of carrageenan (3.92%–4.18%) and alginate (11.04%–11.21%) remained nearly unchanged compared to the control. Protein and ash contents also remained stable, with carrageenan showing 4.25%–4.43% protein and 36.16%–36.25% ash, while alginate showed 0.14%–0.19% protein and 24.89%–26.01% ash. However, viscosity increased proportionally with the dose, from 58.68 cp in the control to 69.78 cp at 7 kGy for carrageenan and from 35.07 cp to 41.87 cp for alginate, suggesting a positive effect on texture‐forming properties. Microbiological evaluation showed a sharp decline in total aerobic bacteria from 1.4 × 10^5^ CFU/g to 0 in carrageenan and from 1.7 × 10^4^ CFU/g to 0 in alginate as doses increased to 7 kGy (Kadir et al. 2023). Therefore, experiments suggest that significant control of microbial load is prominent in the irradiation process, but doses need to be maintained to avoid functional alteration.

Advanced preservation technologies are identified to have different performances in the main mechanism and preservation effects, and among the techniques, HPP has broader applicability in quality preservation and microorganism stabilization.

## 9 Consumer Acceptance & Market Trends of Seaweed

Consumers typically view environmental factors as supplementary advantages rather than the main determinant when selecting food substitutes (Renner et al. 2012). According to Siegrist and Hartmann (2019), hedonic elements, including price, look, and taste, continue to have the greatest influence on decisions. In the case of seaweed products, traditional preservation techniques like boiling or sun‐drying frequently compromise sensory and compositional qualities, such as taste and ingredients, which are significant determinants of consumer preference (Palmieri and Forleo 2020). On the other hand, consumers are favoring new techniques like vacuum packaging, modified atmosphere packaging (MAP), and freeze‐drying because of their capacity to preserve nutritional value and freshness. Technologies like irradiation and nanotechnology, which are sometimes regarded with suspicion despite their efficacy in germ control and nutrient preservation, nonetheless face difficulties in customer perception (Moreira‐Leite et al. 2023; Rabiepour et al. 2024). The high nutrient content and bioactive components of edible seaweeds offer a promising alternative to provide numerous health benefits (Gullón et al. 2020; Milinovic et al. 2021). For example, 76% of participants indicated that they would be happy to consume seaweed, and about 12% of the more than 1000 participants said that they favored snacks made from seaweed (Palmieri and Forleo 2020). The majority of seaweed consumers are young men (Milinovic et al. 2021) because seaweed and fish have similar flavors, people feel more at ease, which may lessen their fear of unfamiliar foods. According to Losada‐López et al. (2021), 57% of respondents said they had prior experience eating seaweed, suggesting that familiarity has a big role.

As seaweed can be found in restaurant recipes and in grocery store dishes, it has become more popular in areas like California and Hawaii, where there are greater Japanese communities. In fact, seaweeds are utilized in Germany and Austria to make a highly sought‐after bread called algenbrot, which is a mixture of cereals that contains up to 3% seaweed. In Brittany, chopped seaweed in butter is used to fry fish or spread on bread to go with shellfish, while dulse and kombu are used to make “bread of the sea” (Cornish 2019).

226 seaweed‐based goods, which are consumed by people, were found in 29 UK stores, comprising 17 high street retailers and accounting for 82.2% of the grocery market share. Of the 224 products that were ultimately examined, 70% were offered in specialty stores, and 30% were found in supermarkets. 25% of the products had no origin label, with the UK accounting for 63% of all origins, Japan for 9%, and China, New Zealand, Thailand, and Switzerland for smaller proportions. The median container size was 134 g, while the median product price was £4.00 (range £0.69–£55.00). These goods fit within the ten primary categories listed in Table 7.

**TABLE 7:** Seaweed‐containing products in each category.

Product category	No. of products (*n* = 224)	% of products	% Retail in supermarkets	Product examples	Reference
Confectionery	42	19	0	Bread, cake, pizza base, biscuits, shortbread	Bouga and Combet (2015)
Condiments	43	19	7	Seaweed flakes, salad booster, salt	
Drinks	5	2	20	Gin, whisky, sugar kelp, smoothie	
Noodles and pasta	9	4	33	Sea spaghetti, kelp noodles	
Salads	7	3	0	Seaweed salad, sea salad	
Seaweed	52	23	14	Whole seaweed, seaweed sheets	
Snacks	34	15	24	Crackers, rice crackers, oatcakes	
Soup	15	7	100	Miso soup	
Supplements	11	5	0	Tablets	
Sushi	5	2	100	Sushi platters	
Other	1	0	0	Gelling agent	

**FIGURE 4:**
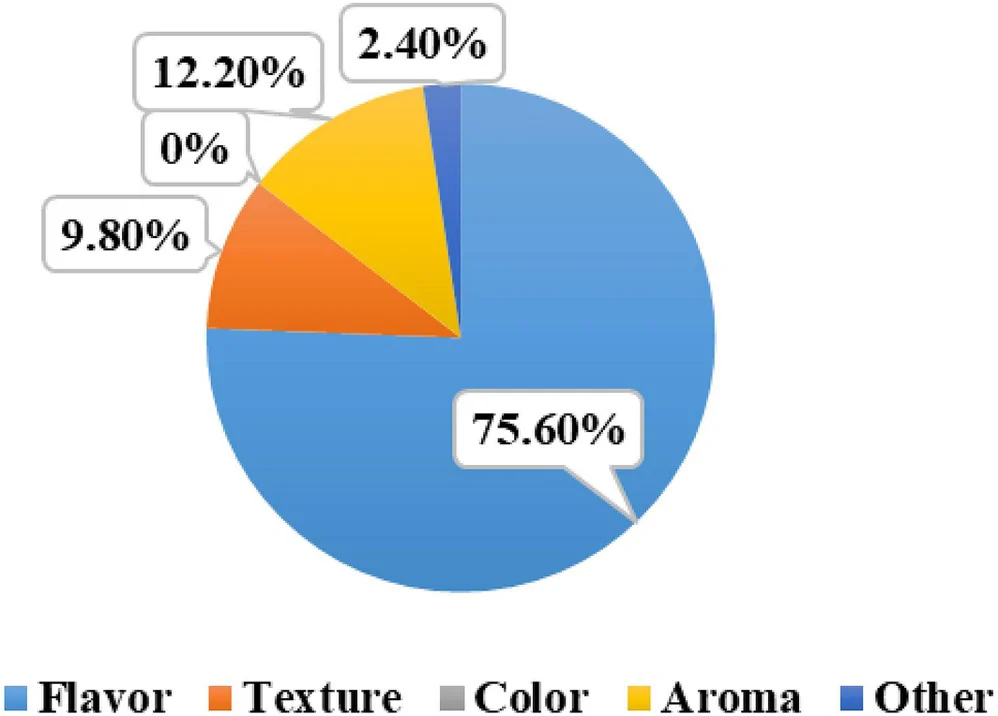
Most important sensory characteristic of seaweed is most important to customer (Arya et al. 2024).

**FIGURE 5:**
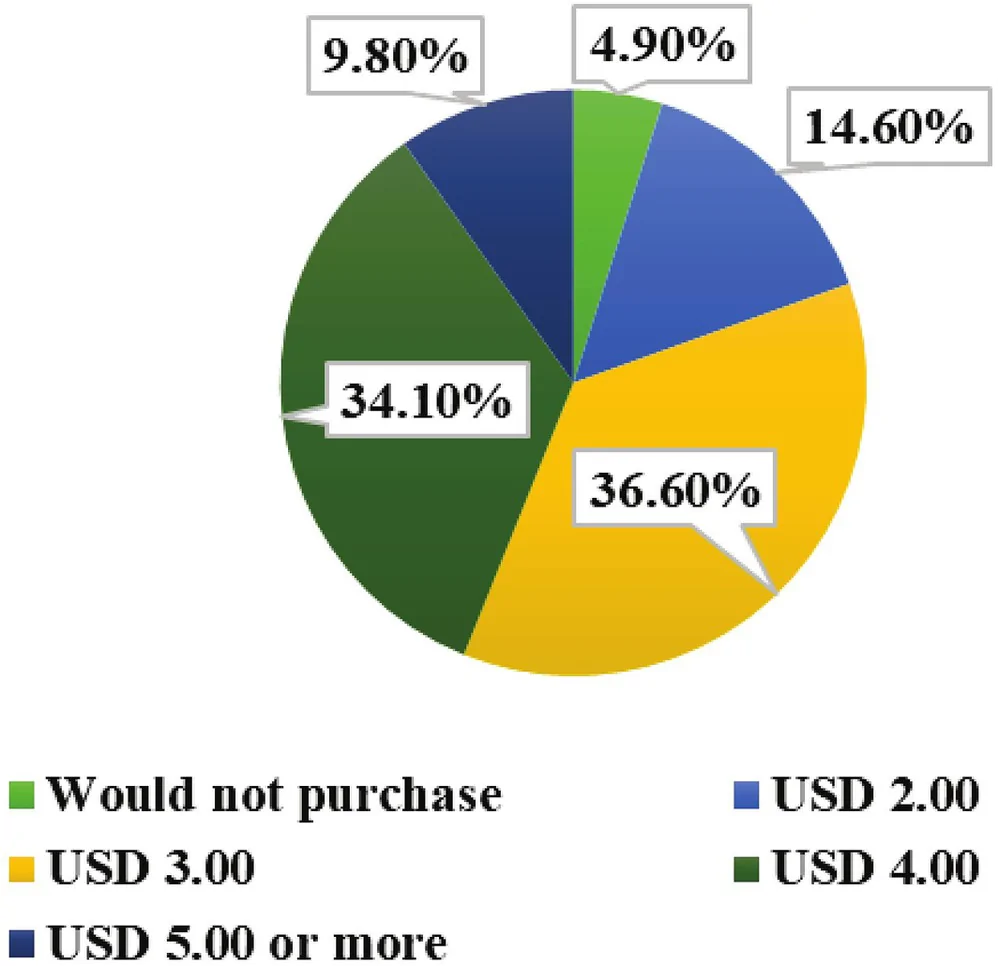
Consumer willingness to pay for a ready‐to‐eat seaweed salad bowl (Arya et al. 2024).

The people surveyed reported that they most frequently ate seaweed as a component of other foods, like sushi (53.7%), followed by salad (17%), soup (9.8%), roasted or mildly toasted seaweed snacks (4.9%), and other foods. Regretfully, many of the panelists reported eating seaweed only once every two to three months. The significance of diversifying seaweed products for the U.S. market is demonstrated by the fact that, when asked what variables may enhance seaweed use, 53.7% of respondents cited availability as a limiting issue, and 46.3% requested additional information regarding nutritional value, among other reasons (Table 8). Flavor (75.6%) was generally recognized as the most significant sensory attribute of seaweed preferred for consumption, followed by odor (12.2%) and texture (9.8%). Interestingly, none of the respondents selected the attribute “color.” That might be because seaweed is becoming more widely available, which has led to consumers becoming more accustomed to and accepting of seaweed's color (Figure 4). Most respondents were willing to pay between USD 3.00 and USD 4.00 for a four‐ounce, ready‐to‐eat seaweed salad bowl (Figure 5). To determine whether certain sodium content levels are prohibitive for certain consumers, more consumer research is required. The majority of panelists (80.5%) thought seaweed was a nutritious food, but 17% were unsure of its health, indicating that the message on nutritional quality needs to be improved (Arya et al. 2024).

**TABLE 8:** What would make you consume seaweed more often? (*n* = 41).

Factors	No. of responses (%)	Reference
Knowing the nutritional value	19 (46.3)	Arya et al. (2024)
Lower price	11 (26.8)	
More availability	22 (53.7)	
Longer shelf‐life	2 (4.9)	
Sustainably grown	6 (14.6)	
Minimally processed	6 (14.6)	
Sold fresh	4 (9.8)	
Sold in ready‐to‐eat dishes	6 (14.6)	
Grown in maine	6 (14.6)	
Others	2 (4.9)	

In addition to consumer education and accessibility, improving formulation strategies may be important to boost global adoption of seaweed‐based foods.

## 10 Conclusion

Seaweed preservation is not only an idea of enhancing the shelf life but also of maintaining the nutritional properties, microbial safety, and consumer demand. The whole concept indicates clearly that, although the technology has advanced, there is no single particular system that can support the full stability of seaweed during the processing time. Moreover, effectiveness often depends on the duration of processing, species type, temperature used, initial microbial load, and market requirements. So, an integrated approach may provide the most useful solution. Another important thing is that there is no standard regulatory framework for seaweed in developing countries. Without the fixed guidelines, running an international trading system, nutrient labelling during product marketing, and shelf‐life determination are also challenging. Collaborations among researchers, stakeholders, and policymakers are essential to set the standards for the proper preservation of seaweed and seaweed‐based products. However, sustainability considerations should be followed in further innovations. Preservation techniques should be easily accessible, energy efficient, environmentally friendly, feasible, and aligned with food security and climate goals. Overall, seaweed preservation practices must head towards the globally accepted strategies that can secure the aspects of nutrition, health, and industry.

## Author Contributions

**Md. Faisal:** conceptualization, funding acquisition, supervision, writing – review and editing, project administration. **Hrishika Barua:** writing – original draft, writing – review and editing, visualization, software, conceptualization. **Shanjida Islam Shikha:** conceptualization, writing – original draft, formal analysis, resources, visualization, data curation.

## Funding

This work was supported by Chattogram Veterinary and Animal Sciences University (MS Student Research Grant).

## Ethics Statement

The authors have nothing to report.

## Conflicts of Interest

The authors declare no conflicts of interest.

## Data Availability

Data sharing not applicable to this article as no datasets were generated or analyzed during the current study.
